# The use of modelling studies to inform planning of health services: case study of rapidly increasing endoscopy services in Australia

**DOI:** 10.1186/s12913-019-4438-x

**Published:** 2019-08-29

**Authors:** Hannah E. Carter, Dylan Knowles, Timothy Moroney, Gerald Holtmann, Tony Rahman, Mark Appleyard, Nick Steele, Michael Zanco, Nicholas Graves

**Affiliations:** 1 Australian Centre for Health Services Innovation, Queensland University of Technology, 60 Musk Ave, Kelvin Grove, QLD 4059 Australia; 2 Anthrodynamics Simulation Services Australia, Homebush, NSW 2140 Australia; 3 Healthcare Purchasing and System Performance, Queensland Health, 33 Charlotte St, Brisbane, QLD 4001 Australia; 4 Faculty of Medicine & Faculty of Health & Behavioural Sciences, University of Queensland, 288 Herston Rd, Herston, QLD 4006 Australia; 5 The Prince Charles Hospital, Rode Road, Chermside, QLD 4032 Australia; 6 Royal Brisbane and Women’s Hospital, Butterfield St, Herston, QLD 4029 Australia; 7 Healthcare Purchasing and System Performance, Queensland Health, 33 Charlotte St, Brisbane, QLD 4001 Australia; 8 Health systems innovation branch, Queensland Health, 33 Charlotte St, Brisbane, QLD 4001 Australia; 9 Department of Gastroenterology & Hepatology, Princess Alexandra Hospital, 199 Ipswich Rd, Woolloongabba, QLD 4102 Australia

**Keywords:** Endoscopy, Discrete event simulation, Service planning, Cost-effectiveness, Waiting list, Model

## Abstract

**Background:**

Demand for gastrointestinal endoscopy in Australia is increasing as a result of the expanding national bowel cancer screening program and a growing, ageing population. More services are required to meet demand and ensure patients are seen within clinically recommended timeframes.

**Methods:**

A discrete event simulation model was developed to project endoscopy waiting list outcomes for two large metropolitan health services encompassing 8 public hospitals in Australia. The model applied routinely collected health service data to forecast the impacts of future endoscopic demand over 5 years and to identify the level of service activity required to address patient waiting times and meet key policy targets. The approach incorporated evidence from the literature to produce estimates of cost-effectiveness by showing longer term costs and Quality Adjusted Life Years (QALYs) associated with service expansion.

**Results:**

The modelling revealed that doing nothing would lead to the number of patients waiting longer than clinically recommended doubling across each health service within 5 years. A 38% overall increase in the number of monthly procedures available was required to meet and maintain a target of 95–98% of patients being seen within clinically recommended timeframes to the year 2021. This was projected to cost the funder approximately $140 million in additional activity over a 5 year period. Due to improved patient outcomes associated with timely intervention, it was estimated that the increased activity would generate over 22,000 additional QALYs across the two health services. This translated to an incremental cost-effectiveness ratio of $6467 and $5974 per QALY for each health service respectively.

**Conclusions:**

Discrete event simulation modelling provided a rational, data based approach that allowed decision makers to quantify the future demand for endoscopy services and identify cost-effective strategies to meet community needs.

**Electronic supplementary material:**

The online version of this article (10.1186/s12913-019-4438-x) contains supplementary material, which is available to authorized users.

## Background

The use of data in health care is undergoing a revolution. Health services are increasingly utilizing information technology, including electronic health records, to capture accurate and comprehensive information across all levels of patient interactions with the system [1]. Health related apps and wearable technology provide a repository of data, with implications for research still evolving. Also emerging is the potential for Blockchain technology to create immense, decentralized, patient generated health databases that are updated in real time [2]. Although challenges exist, the use of routinely collected administrative health data has the potential to transform health service planning. Specifically, the wealth of available data can provide decision makers with a rich and constantly evolving understanding of the broader system which in turn enhances their ability to plan for and deliver effective and efficient services.

Discrete event simulation (DES) is a modelling technique that is able to capitalize on the value inherent in detailed patient-level data. DES models are typically designed to represent the process flow of a complex system and the way in which individuals move through and interact with other agents in the system [3]. This provides a systematic, transparent and comprehensive approach to applying data and evidence in the decision making process. Over the past decade, DES models have emerged as an increasingly important and powerful tool in health service management and delivery [4]. The use of individual simulations, as well as the incorporation of time to event parameters, mean that DES techniques are uniquely placed to model the effects of capacity constraints and waiting lists [5].

In Australia, waiting lists for public endoscopy services have rapidly increased over the past decade driven by population growth and ageing, the introduction of a National Bowel Cancer Screening Program (NBCSP) [6], the increasing need for ongoing surveillance procedures and the substitution of surgical techniques with endoscopy procedures [7]. This trend is expected to continue over the next several years in line with population trends and the expansion of the NBCSP to cover bi-annual screening for all Australians aged 50 to 74 years by 2020. As a result, many health services are becoming unable to see all patients within the clinically recommended time frames. Addressing this issue was identified as an area of priority by the state health department responsible for funding and delivering public endoscopy procedures in a metropolitan region of South-East Queensland, Australia.

The aim of this study was to develop a DES model to inform endoscopy service planning in two large public health services. The model was designed to forecast the impacts of future endoscopic demand and to identify activity levels required to reduce waiting list times and meet policy targets. In addition, it incorporated estimates of future costs and Quality Adjusted Life Years (QALYs), allowing incremental cost-effectiveness ratios to be produced.

## Methods

A discrete event simulation (DES) model of the endoscopy system was developed using AnyLogic [8] to capture the major patient pathways from referral through to diagnosis and ongoing follow up care. A screenshot of the model structure in AnyLogic is in Additional file 1. The model adopted a timeframe of 5 years and was separately applied to two large, public metropolitan health services referred to hereafter as health service A (HSA) and health service B (HSB). Both were similar in size and nature, with each containing four hospital-based endoscopy units and servicing a community population of approximately 900,000 and 1 million people respectively.

### Model structure

**Fig. 1:**
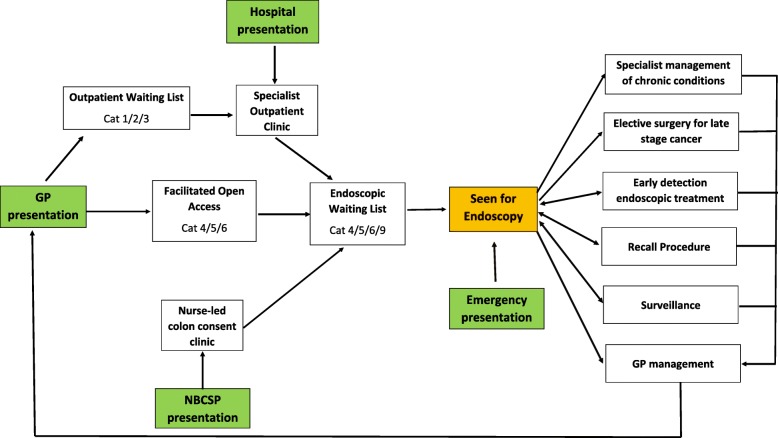
Flow chart describing major patient pathways through a simulation of the endoscopic care system. Legend: Patients could present to the system via general practitioner referrals, the National Bowel Cancer Screening Program (NBCSP), hospital wards and emergency departments. Once assessed, patients were categorized according to their urgency and placed on the endoscopic waiting list if appropriate. Following a period of waiting, patients who did not drop off the waiting list were seen for endoscopy and subsequently allocated to a form of ongoing care which included specialist treatment for chronic conditions, treatment for early or late stage colorectal cancer, a future endoscopic surveillance procedure (rejoining the waiting list as category 9), a recall procedure to be conducted due to inconclusive results. Where no ongoing treatment protocol was indicated, patients transitioned to general practitioner management where they assumed the characteristics of the general population

A visual depiction of the model is in Fig. 1. Category 4 patients were the most urgent and had a 30 day clinically recommended timeframe for being seen by a specialist, while category 5 were recommended to be seen within 90 days and category 6 within 365 days. The type of follow up care patients’ received after an endoscopy was informed by clinical guidelines; this was consistent with service provision and confirmed by a recent internal audit of post-endoscopy management in HSB. Post-endoscopy outcomes were assumed to have implications for patient quality of life, health system costs and mortality rates.

### Data inputs

A comprehensive list of data inputs are in Additional file 2. Data on patient presentations, waiting list outcomes, endoscopy outcomes and costs within the endoscopy system were largely populated with existing administrative data. Where existing data were not available, estimates were based on an audit of hospital data. Estimates of the 5 year survival rates, costs and utility weights associated with post-endoscopy ongoing care states were sourced from the literature.

The Australian health care system is funded by both state and federal budgets, with hospital procedures, including endoscopy services, largely funded by state governments. Costs in this study were valued from the perspective of the state government in recognition of their role as the decision maker in this context.

QALY estimates were derived by multiplying the length of time a patient spent in a given health state, multiplied by the utility weight assigned to that health state. Utility weights are a measure of patient presence on a scale where zero is equivalent to death, and one is equivalent to full health [9]. As utility values were not directly available from the administrative datasets, these were sourced from the published literature and applied to the chronic condition, early bowel cancer and advanced bowel cancer health states. We adopted the simplifying assumption that all other health states in the model had a utility weight equivalent to one.

### Modelled scenarios

A ‘do nothing’ scenario was modelled to provide a baseline estimate of waiting list, cost and QALY impacts over 5 years under the assumption that no changes to existing endoscopy services were implemented. The model then ran a number of simulations to estimate the waiting list impacts associated with specific increases in the weekly number of endoscopic procedures available. Category specific targets were set and the model was used to simulate the capacity and funding levels required to reach these within a given timeframe. Targets were set to achieve 98% of Category 4 patients, and 95% of category 5 and 6 patients, being seen within clinically recommended waiting times. Patients who breached their clinically recommended waiting time are referred to as ‘long waits’.

The modelled results have been reported in terms of increased levels of capacity within the system. It is not assumed that capacity directly correlates with activity levels. Rather, capacity levels reflect the upper limit of possible activity. Activity levels within the model are instead driven by waiting list sizes and demand projections. At various points within the model, activity may be equal to or less than the overall capacity limit. Costs and QALY outcomes are based on the level of activity.

### Key assumptions

The model adopted the annual demand projections provided by the state health department; this included initial presentations via general practitioner, outpatient, NBCSP and emergency settings, as well as subsequent presentations including for surveillance, recall or early detection endoscopic treatment.

Waiting list numbers and waiting times were also modelled to reflect known data, including data on monthly waiting list trends where available. Surveillance patients were classified as category 9 with clinically recommended waiting times that varied between 1 to 5 years as prescribed by national clinical guidelines [10]. Given there was no pre-defined administrative process for prioritising patients on the waiting list, the model ran a number of experiments to determine how to best align the modelled process to known trends. The best fit resulted from a process whereby emergency department patients had immediate priority (reflecting real world practice), followed by categories 4, 5, 6 and 9 patients, prioritized by how close they were to breaching their clinically recommended waiting times. For example, a category 4 patient at 66% of their goal time (i.e. 20 days waiting) was prioritized above a category 6 patient at 50% of their goal time (i.e. 182 days waiting). This was further augmented by a “twenty days” rule whereby patients approaching 20 days before their goal time were more heavily prioritised. While this rule was not an explicit guideline used in waiting list management, we found that it was able to provide the best fit in reflecting real world outcomes. Specifically, it affected category four patients more strongly than other patients due to their short goal time (i.e. 30 days), helping to keep their waiting times more consistently managed as observed in practice.

Despite a prioritisation process that emphasised proximity to clinically recommended waiting times, an element of ‘randomness’ was built into this process in order to more closely reflect reality. As such, patients were not necessarily treated in the order they were added to the waiting list. This prioritisation logic enabled the model to closely mirror observed patient waiting times and long wait trends.

Increased capacity for endoscopy procedures may be attained by either extending the operating hours of existing procedure rooms, building new procedure rooms, or a combination of both. Also required for the expansion of activity levels is a sufficient level of qualified medical and nursing staff, and adequate funding levels to accommodate this. The interplay of these factors varied across each of the individual hospitals and clinics accounted for in the modelling. We therefore determined that accounting for these internal and external constraints on changes to activity levels were beyond the scope of our analysis and more suited to modelling, and decision making, within the individual hospital setting. The analysis presented here therefore does not assume or prescribe to a particular strategy to increase capacity. As a result, costs associated with increasing capacity beyond standard procedure costs (for example, staff overtime or capital costs associated with new equipment or facilities) were excluded from this analysis.

### Model validation

The model was validated through three mechanisms. The first was to closely match well-established waiting list projections and endoscopy costs to the health system under the ‘do nothing’ scenario. The second was to approximate measures, where possible, from a number of different sources. For example, internal feedback loops were validated against historical trend data. This process included running AnyLogic optimization experiments to reduce the differences in simulated waiting list outcomes versus the non-simulated projections. The third mechanism involved a sense check by experts that included a team of health economists, health service managers and senior clinicians who agreed that the model was performing as they would expect and that results were representative of their system. Further information on the validation process, including calibrated measures and margins of error, are reported in Additional file 3.

### Sensitivity analysis

A sensitivity analysis examined the impact of uncertainty in the demand projections. Scenarios where projections were 20% lower and 20% higher than the base case assumption were modelled.

## Results

The base case model predicted an increase in long wait patients by 46% for HSA and 97% for HSB (Table 1) between 2017 and 2021, assuming no increases to the number of endoscopy procedures available.

**Table 1:** Projected increases to long wait patients by category under a ‘do nothing’ scenario

Category	Number of long wait patients						
	Model starting point	2017	2018	2019	2020	2021	% change 2017–2021
Health Service A							
4	#	632	533	486	480	507	−20%
5	#	2154	2396	2603	2942	3347	55%
6	#	360	554	601	654	743	106%
Total	#	3146	3483	3690	4076	4596	46%
Health Service B							
4	#	330	242	217	245	273	−17%
5	#	946	1080	1292	1607	1937	105%
6	#	0	89	162	241	308	–
Total	#	1276	1410	1670	2092	2517	97%

# Data are not publicly available and have been omitted due to their sensitive nature. Figures are below those reported for subsequent years

For HSA, the model identified a tipping point at 480 procedures per week. At this capacity level, patient outcomes began to dramatically improve but improvement was inconsistent across categories and did not allow for clinically recommended wait times to be met within modelled targets. To sustain target clinical waiting times over the course of the 5 year projections, the model estimated that an increase in the number of available procedures by 37% to 560 procedures per week would be necessary. For HSB, a tipping point was found at 480–510 procedures per week, depending on urgency category. An increase of 38%, to 580 procedures per week was required to meet and sustain waiting list targets.

The cost to the funder of increasing capacity levels to those recommended by the modelling was estimated to be $82.9 million over 5 years for HSA, translating to 12,819 QALYs gained and an ICER of $6467 per QALY (Table 2). For HSB, the estimated cost was $60.8 million over 5 years, with 10,177 QALYs gained and an ICER of $5974 per QALY.

**Table 2:** Modelled cost-effectiveness outcomes

Cost-effectiveness outcomes	Health Service A (*N* = 91,564)			Health Service B (*N* = 84,805)		
	Current capacity (410 procedures)	Recommended capacity (560 procedures)	Difference	Current capacity (420 procedures)	Recommended capacity (580 procedures)	Difference
Costs to funder over 5 years ($ Millions)						
Nurse-Led Colon Consent Clinic	*0.44*	*0.5*	*0.1*	*0.5*	*0.5*	*0.0*
Specialist Outpatient Clinic	*8.5*	*8.5*	*0.0*	*8.6*	*8.6*	*0.0*
Facilitated Open Access	*3*	*3*	*0.0*	*3*	*3*	*0.0*
Elective surgery costs	*10.5*	*12.7*	*2.2*	*10.5*	*12.4*	*1.9*
Endoscopy costs: outpatient	*145.7*	*191.8*	*46.1*	*154.7*	*195.8*	*41.1*
Endoscopy costs: inpatient	*108.8*	*142.8*	*34*	*65.1*	*82.6*	*17.5*
Total costs to funder	278.2	361.1	82.9	243.1	303.9	60.8
Average QALYs per patient^a^	2.03	2.17	0.14	2.00	2.12	0.12
Total QALYs	185,875	198,694	12,819	169,610	179,787	10,177
ICER	$6467 per QALY			$5974 per QALY		

*QALY* quality adjusted life years, *ICER* incremental cost-effectiveness ratio

^a^QALYs are recorded only for the time patients spend within the modelled system

A sensitivity analysis revealed that a 20% reduction in projected demand levels would require less than half of the additional capacity and costs estimated under the base case scenario in each health service (Table 3). Conversely, demand levels 20% higher than projected would translate to a total cost increase of 66% across HSA and HSB combined.

**Table 3:** Additional capacity levels and costs associated with changes to demand projections

Modelled outcomes	Base case	Lower estimate	Upper estimate
Health Service A			
Monthly presentations	1150	920	1380
Recommended capacity increase	150	70	240
Health service funder costs over 5 years ($M)^a^	82.9	31.8	136
Health Service B			
Monthly presentations	1200	960	1140
Recommended capacity increase	160	70	240
Health service funder costs over 5 years ($M)^a^	60.8	17	104

^a^Costs relate to additional activity only

## Discussion

This is the first Australian study to apply simulation modelling to inform decision making in endoscopic service planning. The model estimated that a 37 and 38% increase in the number of weekly procedures available was required for HSA and HSB respectively to meet and sustain waiting list targets over 5 years. This translated to an ICER of $6667 per QALY for HSA and $5974 per QALY for HSB. When considering a recent estimate of a reference ICER for the Australian health care system of $28,003, this represents a high value use of resources [11]. This lends support to the evidence that bowel cancer screening is a cost-effective intervention [12, 13].

The use of DES techniques to inform service planning in health care is an emerging field. A 2010 study used a combination of simulation modeling and linear programming with the aim of reducing access times for endoscopy services in a teaching hospital in the Netherlands [14]. The authors reported that more efficient scheduling practices were able to overcome long access times and allow for performance targets to be met. Further, a 2017 study used DES methods combined with demand forecasting in a hybrid approach that aimed to improve the match between demand and capacity [15]. The approach was determined to provide plausible forecasts that could inform future health service capacity planning and resource allocation. DES methods have also been used to evaluate operational performance in a colonoscopy suite, accounting for factors including the number of endoscopists, procedure rooms, and turnaround time [16]. Other recent studies have applied DES modelling to analyse waiting list outcomes in outpatient neurosurgery and orthopedic clinics, as well as for surgical procedures [17–21].

A key strength of our analysis was its ability to model a complex, integrated and multi-facility system. As noted in a 2010 review of DES modelling in health care, such studies are typically unit-specific and model discrete components of broader systems, for example, an emergency department, clinic or operating room. [4] The model presented here comprises a comprehensive, system-level representation that spans multiple referral and assessment points as well a number of post-endoscopy patient health states. An additional strength of the approach we have taken is the use of existing, routinely collected data to populate the model. This has the advantage of saving on both the time and cost associated with prospective data collection, and allows for the model to be constantly updated over time. The identification of clear tipping points in both settings highlighted the potential for this modelling technique to identify the minimum required service level to improve waiting list outcomes in the context of growing demand.

While the flexibility inherent in simulation modelling is one its core strengths, it does require specialized expertise and software that may limit its accessibility to many organisations. The use of individual level data also means that these techniques are often best suited to modelling a specific unit or system, with limitations for generalisability. As such, this analysis should be viewed as a case study that has been tailored to the two health services in question. The results cannot be assumed to apply in other endoscopy services.

There were a number of limitations we encountered with the use of administrative datasets for simulation modelling, a purpose for which it was not originally intended. These data were able to provide good estimates of process based measures, for example waiting list numbers and waiting list movements. However, we identified several gaps in these numbers when extrapolating to overall demand or activity levels. For example, several patients undergo an endoscopy without ever being on the waiting list; this could be following an emergency department presentation or as part of an unplanned hospital admission. These presentations needed to be accounted for separately which involved synthesizing different datasets that were not directly comparable, and augmenting these estimates with clinical opinion. There were other challenges we encountered that may be generalized to administrative health service data more broadly. A major limitation was the lack of patient reported outcome measures, for example quality of life. As a result, our analysis is limited by the simplifying assumption that patients are in a state equivalent to full health for the duration of time they spend waiting to be seen for an endoscopy. Utility values reflecting the health related quality of life for patients with cancer and chronic conditions were taken from the published literature, but it is unknown whether they may differ in this cohort specifically. Long term clinical outcomes including patient survival were also unavailable, further limiting the ability of the model to capture patient health outcomes over the longer term.

Another limitation of the administrative data we used was the use of health service reimbursement rates as a proxy for cost of service provision. There is evidence that the actual costs incurred by the health service may vary across sites [22]. This discrepancy may ultimately inhibit best practice decision making for the sites in question. Our model assumes a constant marginal cost per additional procedure. In practice however, there may be higher marginal costs of additional activity (for example due to staff overtime or capital expenditure on new facilities or equipment), or conversely, lower marginal costs achieved through greater economic efficiencies. Marginal costs depend on the level of latent capacity within the system as well as decision makers’ preferences for managing additional activity; we did not have sufficient information to incorporate these considerations into the model. In addition, we have only accounted for the costs borne by the immediate decision maker, the state health department. This excludes any nationally funded longer term costs associated with ongoing care including pharmaceutical costs, outpatient visits and medical procedures.

Not captured in our model are issues relating to the supply of qualified medical staff if activity levels were to be increased to recommended levels. Sustaining a health workforce that can meet the demands of an ageing population has been identified as a key policy challenge in Australia [23]. A limited supply of endoscopists may have implications for waiting lists beyond what can be addressed through funding for increased activity levels. Further, there is some evidence to suggest that longer procedure times may be associated with higher rates of adenoma detection [24]. These findings are preliminary and need to be examined in further studies, but highlight the potential clinical implications that need to be considered in increasing activity levels within a service.

Despite these limitations, we understand that the outcomes of this study were useful to policy makers. Senior decision makers within the state health department have confirmed that the modelled findings helped inform funding allocations as part of an action plan to improve access to endoscopy and reduce the number of long wait patients. The plan saw the allocation of $160 million over 4 years to increase the number of endoscopy services provided across the state by 50,000.

## Conclusions

Health service decision makers are increasingly faced with financial pressures that reflect growing, ageing populations as well as more costly clinical interventions. In response to concerns around the sustainability of the health care system, there has been a movement towards value base care whereby decision makers aim to maximise health benefits from fixed budgets. The use of data to inform rational decision making with a focus on cost-effectiveness is critical to achieving this aim. The methods we have presented here may have implications for health service managers in making a case for investments in new infrastructure based on forward looking capacity planning, as opposed to small annual funding increments that may contribute to over-burdened services.

Our results demonstrate the power of discrete event simulation in using patient level data to inform health care decision making, specifically in terms of managing demand and improving waiting list outcomes. The model was able to provide a recommendation of the level of service activity required to meet specific policy targets within given timeframes, as well as an estimate of the cost-effectiveness associated with this. This provided decision makers with a rational, data based approach in determining the allocation of scarce health care resources.

## Additional files

Additional file 1: Model structure. Screenshot of the model structure as developed within the Anylogic software program. (JPG 774 kb)

Additional file 2: Model inputs. A comprehensive summary of the modelled input parametes and respective data sources. (PDF 118 kb)

Additional file 3: Validation processes. A detailed description of the model validation process, including calibrated measures and margins of error. (PDF 80 kb)

## Data Availability

The full set of modelled input data is available from the corresponding author on request. Data was sourced from administrative databases including the Queensland Gastrointestinal Endoscopy Data Collection and the Queensland Admitted Patient Data Collection (https://www.health.qld.gov.au/__data/assets/pdf_file/0034/843199/data_custodian_list.pdf).

## Abbreviations

DES: Discrete event simulation
HSA: Health Service A
HSB: Health Service B
ICER: Incremental cost-effectiveness ratio
NBCSP: National Bowel Cancer Screening Program
QALY: Quality adjusted life year
