# Supplementary figures and images for: The use of modelling studies to inform planning of health services: case study of rapidly increasing endoscopy services in Australia
Source: BMC Health Serv Res. 2019 Aug 29;19:608. doi: 10.1186/s12913-019-4438-x (PMC6716875; doi:10.1186/s12913-019-4438-x)

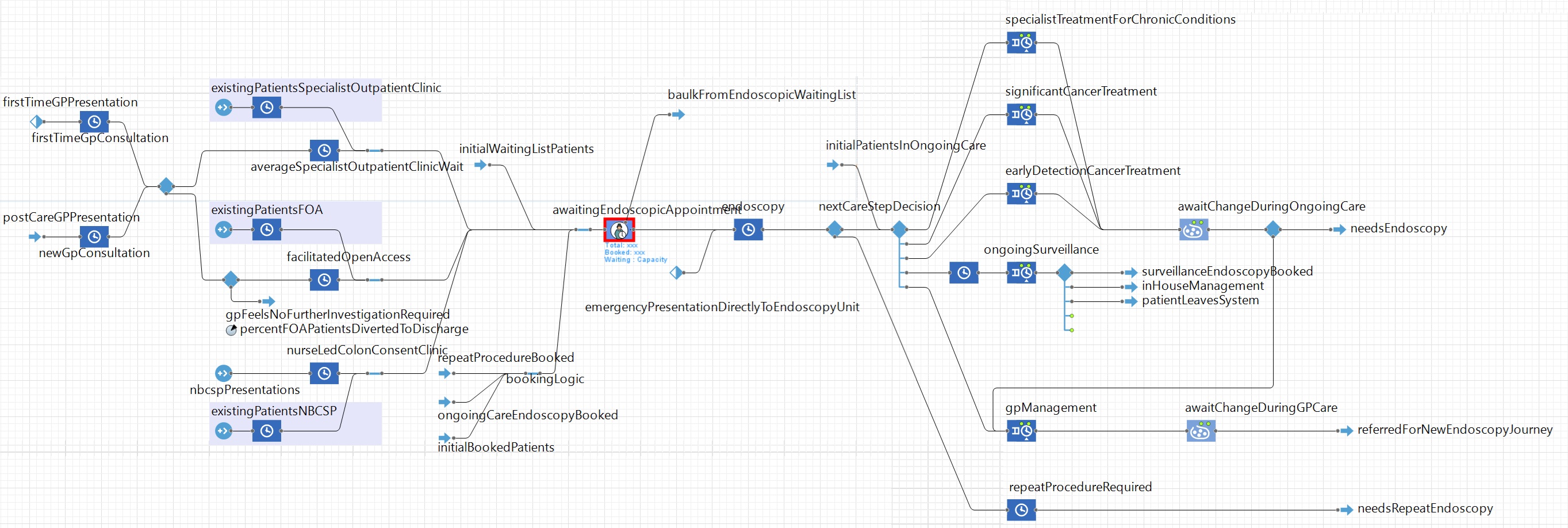

Supplement: Supplementary file 1 — Model structure. Screenshot of the model structure as developed within the Anylogic software program. (JPG 774 kb) [file 12913_2019_4438_MOESM1_ESM.jpg]
