# Supplementary material for: The use of modelling studies to inform planning of health services: case study of rapidly increasing endoscopy services in Australia
Source: BMC Health Serv Res. 2019 Aug 29;19:608. doi: 10.1186/s12913-019-4438-x (PMC6716875; doi:10.1186/s12913-019-4438-x)
Supplement: Supplementary file 2 — Model inputs. A comprehensive summary of the modelled input parametes and respective data sources. (PDF 118 kb) [file 12913_2019_4438_MOESM2_ESM.pdf]

Data inputs

Category	Parameter description	HSA	HSB	Source
Demand projections	2017 (base year)	14,944	12,242	State government demand forecasting*
	2018	16,631	14,598	
	2019	18,315	16,958	
	2020	19,997	19,321	
	2021	21,677	21,686	
Patient presentation to system	Facilitated access	57%		HSA administrative data
	Specialist outpatient	37%		HSA administrative data
	NBCSP	5%		State government NBCSP projections
	Emergency department	1%		Expert opinion, supported by administrative data
Initial waiting list numbers, by category	Category 4	738	504	State government administrative data
	Category 5	2,266	835	
	Category 6	1,188	521	
	**Total**	**4,192**	**1,860**	
Waiting list movements	**Category 4**			State government administrative data
	Patients seen	535	624	
	Patients added	642	796	
	Patients removed	7	5	
	**Category 5**			
	Patients seen	324	321	
	Patients added	423	409	
	Patients removed	18	9	
	**Category 6**			
	Patients seen	50	84	
	Patients added	85	107	
	Patients removed	12	6	

Post-endoscopy outcomes, patients presenting via GP, hospital or ED	Specialist management of chronic conditions	10%	HSB hospital audit data^
	Elective surgery for the treatment of late stage cancer#	1.25%	State government administrative data
	Early detection endoscopic treatment	9%	HSB hospital audit data^
	Recall procedure	3.40%	HSB hospital audit data^
	Surveillance procedure	25%	HSB hospital audit data^
	relative proportion within 3 years	*39% of surveillance*	HSB hospital audit data^
	relative proportion within 5 years	*61% of surveillance*	HSB hospital audit data^
	Ongoing GP management	~ 51%	Remaining proportion
Post-endoscopy outcomes, patients presenting via NBCSP	Specialist management of chronic conditions	10%	HSB hospital audit data^
	Elective surgery for the treatment of late stage cancer[>]	0.80%	Adjusted risk based on Doubeni et al [19]
	Early detection endoscopic treatment	7%	Adjusted risk based on Doubeni et al [19]
	Recall procedure	3.40%	HSB hospital audit data^
	Surveillance procedure	0%	NA[#]
	Ongoing GP management	~ 79%	Remaining proportion
Costs	Outpatient clinic	285	State government reimbursement item cost [20]
	Facilitated access	234	State government reimbursement item cost [20]
	Nurse led colon consent clinic	234	State government reimbursement item cost [20]
	Inpatient endoscopic procedures	6,700	State government administrative data
	Outpatient endoscopic procedures	1,585	State government price weight [20]
	Elective surgery for late stage colorectal cancer	16,854	State government administrative data
	All other ongoing care states	0	Simplifying assumption
5 year survival rates	Early stage bowel cancer	0.875	Heitman et al [21]
	Late stage bowel cancer	0.335	Heitman et al [21]
	All other states	Varies by age	Australian life tables [22]
Health state utility values	Specialist treatment for chronic conditions (severe IBD)	0.72	Gibson et al [23]
	Advanced bowel cancer	0.74	Heitman et al [21]
	Early bowel cancer	0.46	Heitman et al [21]
	All other health states	1	Simplifying assumption

HSA = health service A; HSB = health service B; NBCSP = National Bowel Cancer Screening Program; GP = general practitioner; ED = emergency department.

*Based on historic demand trends with an additional allowance for the increase in NBCSP presentations

^Audit on endoscopy outcome data from two hospitals based in HSB

#Surveillance procedures for NBCSP patients are accounted for in demand projections

~ These figures are an approximation derived as a function of the remaining proportion after transitions to other heath states have been accounted for.
