# Supplementary material for: The use of modelling studies to inform planning of health services: case study of rapidly increasing endoscopy services in Australia
Source: BMC Health Serv Res. 2019 Aug 29;19:608. doi: 10.1186/s12913-019-4438-x (PMC6716875; doi:10.1186/s12913-019-4438-x)
Supplement: Supplementary file 3 — Validation processes. A detailed description of the model validation process, including calibrated measures and margins of error. (PDF 80 kb) [file 12913_2019_4438_MOESM3_ESM.pdf]

Model validation process

The model was validated through three mechanisms. The first was to closely match well-established waiting list projections and endoscopy costs to the health system in the business-as-usual scenario. In the model, health service A (HAS) and health service B (HSB) waiting list projections were approximated from 2017 to 2021. All calibrations aimed to match these projections within 10% error, allowing for deviation closer to 2021 as unknowns and model factors, such as the rate of patients dropping off the waiting list and the return of category nine (surveillance) patients, would cause the model to drift from the projected linear trend. Within this 10% error bound calibrations were made such that category four, five, and six waiting list sizes approximated their respective demand projections. These were not required to match within 10% error to avoid overfitting: tuning the model to tightly match the relatively small count of category four patients, for example, would detriment the accuracy of category five and six and vice versa. Category nine was used as a guide but not a rule due to the known inaccuracy of the underlying data. In the model, costs to the health system were disaggregated by presentation source to the endoscopy unit but, as only aggregate trends existed, were validated against the total annual cost to the Queensland health system. As in the status quo the number of endoscopies per week were effectively fixed, the cost was also assumed to be fixed each year. The acceptable error bound for this figure was placed at 25% as, due to random variation in the costs assigned to patients receiving endoscopies, this figure was more apt to vary between model runs.

The second validation mechanism was to approximate supporting measures, such as the number of long wait patients and proportion arriving to the endoscopy system from different sources. As per waiting list projections, long wait patients were broken into category four, five, and six patients. Only historical trends were known for long wait patients: as their growth was roughly linear if outlier years were ignored (e.g., category four long wait patient counts jumped from 497 individuals to 1194 individuals between February and March 2016, with a return to stable trends thereafter), model calibrations were given more credence if long wait patient counts grew at a linear rate roughly in line with historical data. Similarly, the proportion of patients on the endoscopy waiting list from each presentation source (e.g., Faciltated Open Access vs GP vs return patient presentation) and the number of patients in specific states, such as the number of people undergoing significant late-stage cancer treatment, were monitored to ensure calibrations did not make valid projections for primary measures due to incorrect processes that would negatively impact secondary measures. More plainly, these measures were used to help ensure that the model was not getting the right answer for the wrong reasons.

The third validation mechanism was a sense check by experts. Model results were passed to the domain expert team of health economists, health service managers and clinicians, who examined the outcomes for mismatches with known figures and expert intuition. While this is a critical component to any validation exercise, the physical separation between modeller and expert team in this instance meant that knowledge only known to those on site could be used to sense check the model.
